# Cumulative risk factors for flap failure, thrombosis, and hematoma in free flap reconstruction for head and neck cancer: a retrospective nested case-control study

**DOI:** 10.1097/JS9.0000000000002069

**Published:** 2024-09-05

**Authors:** Pei-Hsin Hsiung, Ho-Yin Huang, Wei-Yu Chen, Yur-Ren Kuo, Ying-Chi Lin

**Affiliations:** a Master Program in Clinical Pharmacy, School of Pharmacy, College of Pharmacy, Kaohsiung Medical University; b School of Pharmacy, College of Pharmacy, Kaohsiung Medical University; c Department of Pharmacy, Kaohsiung Medical University Hospital, Kaohsiung Medical University; d Division of Plastic Surgery, Department of Surgery, Kaohsiung Medical University Hospital, Kaohsiung Municipal Ta-Tung Hospital; e Faculty of Medicine, College of Medicine, Kaohsiung Medical University; f Department of Biological Sciences, National Sun Yat-Sen University; g Master/Doctoral Degree Program in Toxicology, College of Pharmacy, Kaohsiung Medical University, Kaohsiung, Taiwan; h Academic Clinical Programme for Musculoskeletal Sciences, Duke-NUS Graduate Medical School, Singapore

**Keywords:** free flap, head and neck cancer, hematoma, risk factors, thrombosis

## Abstract

**Background:**

Free flap construction enhances the quality of life for head and neck cancer (HNC) patients; however, complications, such as thrombosis and hematoma, threaten flap survival. This study aimed to identify factors influencing flap failure, thrombosis, and hematoma.

**Methods:**

A retrospective nested case-control study was conducted on HNC patients who underwent free flap reconstruction at a tertiary medical center between January 2019 and January 2022. All patients received antithrombotic prophylaxis consisting of prostaglandin E1, dextran, aspirin, and dipyridamole. Risk factors were analyzed using multivariate logistic regression.

**Results:**

Among 548 flaps analyzed, flap failure, thrombosis, and hematoma rates were 4.74%, 3.83%, and 9.65%, respectively. Risk factors for flap failure included thrombosis (OR 86.42, 95% CI 15.73–474.89), smoking (OR 49.44, 95% CI 1.28–>1000), posteromedial thigh (PMT) flap usage (OR 14.05, 95% CI 2.48–79.54), hematoma (OR 9.68, 95% CI 2.35–39.79), and younger age (OR 0.93, 95% CI 0.87–0.99). Thrombosis risk factors included PMT usage (OR 11.45, 95% CI 2.60–50.38) and anastomosis with the superior thyroid vein (SThV) as the recipient vein after multiple reconstructions (OR 7.91, 95% CI 2.06–30.39). Hematoma risk factors included fibula osteocutaneous flap usage (OR 9.22, 95% CI 2.71–31.42), double-flap usage (OR 8.88, 95% CI 1.80–43.81), liver cirrhosis (OR 6.28, 95% CI 1.44–27.47), and post-surgery hypertension (OR 2.77, 95% CI 1.39–5.50), whereas ipsilateral recurrence (OR 0.14, 95% CI 0.03–0.73) and using the external jugular vein (EJV) as the recipient vein (OR 0.22, 95% CI 0.08–0.61) were protective factors.

**Conclusion:**

Thrombosis poses a greater risk than hematoma for flap failure. Utilization of the PMT flap and the SThV markedly increased the risk of thrombosis and flap failure. These findings highlight the importance of antithrombotic prophylaxis and the selection of flaps and recipient veins in recurrent HNC patients.

## Introduction

Highlights

- Thrombosis, smoking, hematoma, posteromedial thigh flap after multiple reconstructions and younger age increase the risk of failure.
- Thrombosis is a more serious complication than hematoma in cases of flap failure.
- Multiple reconstructions using the posteromedial thigh flap and anastomosis with the superior thyroid vein as the recipient vessel elevate the risk of thrombosis.
- Liver cirrhosis, fibula osteocutaneous flap, double-flap, and postsurgical hypertension are associated with hematoma formation.
- Optimal flap and recipient vein selection for reconstruction and antithrombotic prophylaxis are the key factors to prevent flap failure.

Free flap reconstruction is the most common procedure following cancer ablation for patients with head and neck cancer (HNC); this procedure can restore crucial functions such as breathing, swallowing, and speech and improve esthetic appearance^1^. Postoperative local complications in free flap surgery, such as thrombosis and hematoma, can compromise flap survival. Thrombosis, the formation of venous clots that block blood flow, occurs most commonly within 48 hours after head and neck free flap surgery^2–4^. Hematoma, a hemorrhage from the surrounding tissues, or injured blood vessels that result in vessel compression occurs mostly within the first week^5,6^. The administration of antithrombotic agents may prevent the formation of thrombosis, but these agents may adversely increase the risk of hematoma^7–15^. There is no consensus regarding whether antithrombotic prophylaxis should be routinely administered.

Studies have indicated that patient characteristics including age, smoking, obesity, diabetes, and hypertension may play a role in flap failure^16,17^. Surgical aspects such as prolonged surgery time, flap type, prior surgery or radiation at the anastomosis site, and choice of recipient vein have also been suggested to be risk factors for flap failure^16,18,19^. However, the risk factors specific to thrombosis and hematomas have been less studied.

To better personalize care for head and neck cancer patients after free flap surgery, we designed a nested case-control study to explore the risk factors for thrombosis, hematoma, and flap failure in patients who underwent free flap surgery for head and neck cancer.

## Methods

A retrospective case-control study was conducted on patients with HNC who underwent free flap surgery at a tertiary medical center in southern Taiwan from January 2019 to January 2022. Free flap reconstruction was performed by a well-trained microsurgery team. Patient information was retrospectively obtained from the electronic medical records. This study was approved by the hospital ethics committee and registered at ClinicalTrials.gov. Given the retrospective nature of this study, the need to obtain informed consent was waived. The identification of malignant head and neck neoplasms was based on the International Classification of Diseases, Tenth Revision (ICD–10), including the codes C00–C14, C31, C32, C73, C75.0, C75.4, and C41.1, according to a previous study^20^. Our standard postoperative antithrombotic prophylaxis protocol consisted of intravenous dextran-40 (10%) and prostaglandin E1 (PGE1) 80 mcg per 500 ml at 21 ml/h for the first 3–5 days, aspirin 100 mg per day for 1–2 weeks and dipyridamole 25 mg three times per day for ~2–4 weeks. A discontinuation interval of 72 h was used for elderly patients or patients already treated with anti-aggregant therapy or anti-coagulation. The dosage of antithrombotic prophylaxis was monitored by routine screening coagulation tests such as the prothrombin time (PT), activated partial thromboplastin time (APTT) and international normalized ratio (INR), after surgery. Patients resumed the original oral anti-aggregant or anti-coagulant 3–5 days postoperatively uneventfully. Most patients underwent temporary tracheostomy. Patients who underwent neck lymph node dissection were intubated and transferred to the ICU under routine sedation within 1–2 days postoperatively until extubation to prevent neck bleeding or hematoma-compromised airway and flap circulation. The postoperative blood glucose levels of the patients were controlled within 150–200 mg/dl by insulin. Patients who underwent flap surgeries without HNC diagnosis codes as the surgical indication, who had benign neoplasms, who were missing surgical charts, or who were not receiving standard antithrombotic prophylaxis therapy were excluded. Each flap surgery was considered an independent case to measure outcomes.

Data concerning cancer stage, alcohol use, betel nut intake, smoking status, and baseline comorbidities were obtained from patient records three days before the operation. Lifestyle was divided into current and past users (yes) and never (no). Comorbidities, such as hypertension, diabetes, coronary heart disease, stroke history, chronic kidney disease with creatinine clearance less than 30 ml/min, and cirrhosis, were extracted from medical records. Prior therapy, including chemotherapy and radiotherapy, and the previous free flap history of the recipient site for HNC (contralateral or ipsilateral recurrence) were confirmed from the cancer registry and past operation records. Surgical characteristics including ablation time, reconstruction time, donor sites, recipient sites, neck dissection, flap size, anastomosis, artery type, vein type, and intraoperative sealant use were retrieved from the operation records. Patients with two flaps sharing a unique pedicle were sorted into the double-flap group. Patients with a single flap other than the anterolateral thigh flap; fibula osteocutaneous flap; radial forearm flap; tensor fascia latae flap; posteromedial thigh (PMT) flap, also referred to be the profunda femoral artery perforator (PAP) flap; and lower medial thigh (LMT) flap were classified as ‘other’. The flap size was calculated by multiplying the flap length and width in the operation record. The type of single artery in each patient included the superior thyroid, carotid, superficial temporal, and facial arteries. One or two veins were used in the center; thus, vein type was regarded as an independent variable. The vein types recorded included the internal jugular vein branch, external jugular vein (EJV), facial vein, and superior thyroid vein (SThV). Intraoperative hemostasis was defined as the administration of fibrin sealant (Tisseel) or a hemostatic matrix (FloSeal) during the operation. Postsurgical hypertension was defined as the use of intravenous antihypertensive medication to control hypertension after surgery^21^. The calcium channel blocker nicardipine was used in our hospital. Medical records were collected for up to 30 days postoperatively.

Reoperation was performed when patients experienced major complications refractory to bedside management. In this study, cases were defined as those with a surgical indication for reoperation due to complications, namely failure, thrombosis, or hematoma within 30 days postoperatively. Patients without complications within the cohort were defined as controls.

Logistic regression analysis was performed to analyze the associations between the outcomes and the variables. Missing values in the flap length or width were filled with the average size of the patient’s cancer location. For multivariate logistic regression, variables with *P* less than 0.2 at the univariate level were selected for inclusion in the model. Adjusted odds ratios (aORs) and 95% CIs were derived for each independent risk factor. The overall statistical significance was set at *P* less than 0.05. All the statistical analyses were performed using SAS Enterprise Guide 8.3 software. The manuscript was prepared according to the STROCSS 2021 criteria^22^.

## Results

**Figure 1:**
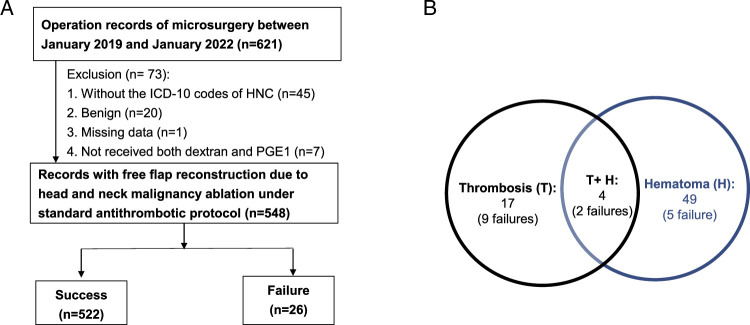
Patient selection flow chart (A) and the thrombosis/hematoma complications (B). HNC, head and neck cancer; ICD-10, International Classification of Diseases, Tenth Revision; PGE1, prostaglandin E1.

In total, 548 flaps from 540 patients were included in this study (Fig. 1). The flap failure rate was 4.74% (26/548). The incidence rates of thrombosis and hematoma were 3.83% (21/548) and 9.65% (53/548), respectively. Flap failure after thrombosis and hematoma were observed in 52.38% (11/21) and 13.21% (7/53) of patients, respectively. The lips, oral cavity, and mouth (499/548, 91.1%) were the most common locations of malignant tumors.

The demographics and baseline characteristics of the patient population and flap surgeries stratified by flap failure, thrombosis, and hematoma are presented in Table 1 and Table 2, respectively. The patient population was mainly male (516/548, 94.16%), with a mean age of 58.4 years, late-stage HNC (452/548, 82.48%), betel nut chewing (415/548, 75.73%), drinking (351/548, 64.05%), and smoking (452/548, 82.48%). In this series, the ALT flap (453/548, 82.67%) was the most common donor site. The superior thyroid artery (408/548, 74.45%) was the most common recipient artery, whereas the internal jugular vein (IJV) branch (408/548, 74.45%) was the most commonly used vein. The posteromedial thigh (PMT) flap, also known as the profunda femoral artery perforator (PAP) flap, and the superior thyroid vein (SThV) as the recipient vessel were only used after multiple reconstructions when limited options were available.

**Table 1:** Baseline characteristics.

	Total (*n*=548)	Failure (*n*=26)		Thrombosis (*n*=21)		Hematoma (*n*=53)	
Variables	*N* (%), mean (SD)	*N* (%), mean (SD)	OR (95% CI)	*N* (%), mean (SD)	OR (95% CI)	*N* (%), mean (SD)	OR (95% CI)
Sex							
Male	516 (94.16)	24 (4.65)	[Reference]	20 (3.88)	[Reference]	51 (9.88)	[Reference]
Female	32 (5.84)	2 (6.25)	1.37 (0.31–6.06)	1 (3.13)	0.80 (0.10–6.16)	2 (6.25)	0.61 (0.14–2.62)
Age	58.4 (9.52)	54.7 (9.25)	0.96 (0.92–1.00)	55.1 (8.35)	0.96 (0.92–1.01)	58.7 (10.54)	1.00 (0.97–1.03)
BMI, kg/m^2^	23.6 (4.09)	24.7 (5.39)	1.07 (0.97–1.17)	25.3 (4.94)	1.10 (1.00–1.22)	23.9 (3.89)	1.02 (0.95–1.09)
Stage							
Early	96 (17.52)	5 (5.21)	[Reference]	6 (6.25)	[Reference]	9 (9.38)	[Reference]
Late	452 (82.48)	21 (4.65)	0.89 (0.33–2.41)	15 (3.32)	0.52 (0.19–1.36)	44 (9.74)	1.04 (0.49–2.22)
Alcohol							
Yes	351 (64.05)	15 (4.27)	0.76 (0.34–1.68)	14 (3.99)	1.13 (0.45–2.84)	40 (11.40)	1.82 (0.95–3.49)
No	197 (35.95)	11 (5.58)		7 (3.55)		13 (6.60)	
Betel nut							
Yes	415 (75.73)	19 (4.58)	0.86 (0.36–2.10)	16 (3.86)	1.03 (0.37–2.86)	37 (8.92)	0.72 (0.38–1.33)
No	133 (24.27)	7 (5.26)		5 (3.76)		16 (12.03)	
Smoking							
Yes	452 (82.48)	25 (5.53)	5.56 (0.74–41.54)	18 (3.98)	1.29 (0.37–4.45)	44 (9.74)	1.04 (0.49–2.22)
No	96 (17.52)	1 (1.04)		3 (3.13)		9 (9.38)	
Pre-chemotherapy							
Yes	120 (21.90)	7 (5.83)	1.33 (0.55–3.25)	2 (1.67)	0.37 (0.08–1.59)	10 (8.33)	0.81 (0.40–1.67)
No	428 (78.10)	19 (4.44)		19 (4.44)		43 (10.05)	
Pre-radiotherapy							
Yes	126 (22.99)	8 (6.35)	1.52 (0.65–3.59)	3 (2.38)	0.55 (0.16–1.89)	10 (7.94)	0.76 (0.37–1.56)
No	422 (77.01)	18 (4.27)		18 (4.27)		43 (10.19)	
Hypertension							
Yes	216 (39.42)	14 (6.48)	1.85 (0.84–4.08)	11 (5.09)	1.73 (0.72–4.14)	23 (10.65)	1.20 (0.68–2.13)
No	332 (60.58)	12 (3.61)		10 (3.01)		30 (9.04)	
Diabetes							
Yes	136 (24.82)	6 (4.41)	0.91 (0.36–2.30)	7 (5.15)	1.54 (0.61–3.91)	18 (13.24)	1.64 (0.90–3.01)
No	412 (75.18)	20 (4.85)		14 (3.40)		35 (8.50)	
Coronary heart disease							
Yes	24 (4.38)	1 (4.17)	0.87 (0.11–6.69)	NA	—	1 (4.17)	0.40 (0.05–2.98)
No	524 (95.62)	25 (4.77)		21 (4.01)		52 (9.92)	
Stroke history							
Yes	15 (2.74)	1 (6.67)	1.45 (0.18–11.48)	1 (6.67)	1.83 (0.23–14.63)	NA	—
No	533 (97.26)	25 (4.69)		20 (3.75)		53 (9.94)	
Severe CKD^a^							
Yes	22 (4.02)	2 (9.09)	2.09 (0.46–9.47)	NA	—	5 (22.73)	2.93 (1.04–8.29)
No	526 (95.99)	24 (4.56)		21 (3.99)		48 (9.13)	
Cirrhosis							
Yes	11 (2.01)	1 (9.09)	2.05 (0.25–16.63)	NA	—	4 (36.36)	5.69 (1.61–20.13)
No	537 (97.99)	25 (4.66)		21 (3.91)		49 (9.13)	

CKD, chronic kidney disease; NA, not applicable; OR, odds ratio.

^a^ It was defined as creatinine clearance <30 ml/min.

**Table 2:** Characteristics related to the flap surgery.

	Total (*n*=548)	Failure (*n*=26)		Thrombosis (*n*=21)		Hematoma (*n*=53)	
Variables	*N* (%), mean (SD)	*N* (%), mean (SD)	OR (95% CI)	*N* (%), mean (SD)	OR (95% CI)	*N* (%), mean (SD)	OR (95% CI)
Free flap history							
None	419 (76.46)	17 (4.06)	[Reference]	18 (4.30)	[Reference]	44 (10.50)	[Reference]
Contralateral recurrence	48 (8.76)	6 (12.50)	3.38 (1.26–9.03)	3 (6.25)	1.49 (0.42–5.24)	7 (14.58)	1.46 (0.62–3.44)
Ipsilateral recurrence	81 (14.78)	3 (3.70)	0.91 (0.26–3.18)	NA	—	2 (2.47)	0.22 (0.05–0.91)
Neck dissection							
Yes	413 (75.36)	19 (4.60)	0.88 (0.36–2.15)	18 (4.36)	2.01 (0.58–6.92)	37 (8.96)	0.73 (0.39–1.36)
No	135 (24.64)	7 (5.19)		3 (2.22)		16 (11.85)	
Ablation time, h	5 (0.35)	4.9 (0.36)	0.70 (0.24–2.08)	5.0 (0.32)	1.00 (0.28–3.50)	5.0 (0.30)	0.94 (0.42–2.11)
Reconstruction time, h	6 (0.36)	5.9 (0.39)	2.09 (0.77–5.68)	5.9 (0.40)	2.32 (0.78–6.94)	5.7 (0.36)	0.89 (0.40–1.98)
Donor site							
Anterolateral thigh	453 (82.70)	17 (3.75)	[Reference]	14 (3.09)	[Reference]	40 (8.83)	
Fibula	22 (4.00)	NA	—	1 (4.55)	1.49 (0.19–11.84)	6 (27.27)	3.85 (1.43–10.40)
Forearm	5 (0.90)	NA	—	NA	—	NA	—
Tensor fascia lata	17 (3.10)	NA	—	1 (5.88)	1.95 (0.24–15.76)	2 (11.76)	1.37 (0.30–6.21)
Posteromedial thigh	29 (5.30)	7 (24.14)	6.38 (2.31–17.65)	4 (13.79)	4.80 (1.48–15.62)	1 (3.45)	0.35 (0.05–2.67)
Lower medial thigh	7 (1.30)	NA	—	NA	—	NA	—
Other^a^	5 (0.90)	1 (20.00)	12.77 (2.19–74.58)	NA	—	1 (20.00)	2.06 (0.23–18.02)
Double^b^	10 (1.80)	1 (10.00)	2.84 (0.34–23.68)	1 (10.00)	3.47 (0.41–29.29)	3 (30.00)	4.40 (1.10–17.70)
Flap size, cm^2^	104 (68.63)	127.6 (94.96)	1.00 (1.00–1.01)	127.5 (106.25)	1.00 (1.00–1.01)	108.9 (79.69)	
Recipient artery							
Superior thyroid artery	408 (74.45)	16 (3.92)	[Reference]	15 (3.68)	[Reference]	37 (9.07)	[Reference]
Facial artery	66 (12.04)	1 (3.23)	0.82 (0.11–6.37)	NA	—	6 (19.35)	2.41 (0.93–6.24)
Carotid artery	31 (5.66)	NA	—	1 (10.00)	2.91 (0.35–24.48)	NA	—
Superficial temporal artery	10 (1.83)	6 (9.09)	2.45 (0.92–6.51)	2 (3.03)	0.82 (0.18–3.67)	6 (9.09)	1.00 (0.41–2.48)
Other arteries	33 (6.02)	3 (9.09)	2.45 (0.68–8.88)	3 (9.09)	2.62 (0.72–9.56)	4 (12.12)	1.38 (0.46–4.15)
Vein number							
One	217 (39.60)	14 (6.45)	[Reference]	9 (4.15)	[Reference]	24 (11.06)	[Reference]
Two	331 (60.40)	12 (3.63)	0.55 (0.25–1.20)	12 (3.63)	0.87 (0.36–2.10)	29 (8.76)	0.77 (0.44–1.37)
Internal jugular vein branch							
Yes	408 (74.45)	17 (4.17)	0.63 (0.28–1.45)	16 (3.92)	1.10 (0.40–3.07)	42 (10.29)	1.35 (0.67–2.69)
No	140 (25.55)	9 (6.43)		5 (3.57)		11 (7.86)	
External jugular vein							
Yes	134 (24.45)	2 (1.49)	0.25 (0.06–1.06)	5 (3.73)	0.96 (0.35–2.68)	7 (5.22)	0.44 (0.19–1.00)
No	414 (75.55)	24 (5.80)		16 (3.87)		46 (11.11)	
Facial vein							
Yes	27 (4.93)	NA	—	NA	—	3 (11.11)	1.18 (0.34–4.05)
No	521 (95.07)	26 (4.99)		21 (4.03)		50 (9.60)	
Superior thyroid vein							
Yes	32 (5.84)	4 (12.50)	3.21 (1.04–9.94)	4 (12.50)	4.19 (1.32–13.30)	2 (6.25)	0.61 (0.14–2.62)
No	516 (94.16)	22 (4.26)		17 (3.30)		51 (9.88)	
Other veins^c^							
Yes	73 (13.32)	5 (6.85)	1.59 (0.58–4.36)	2 (2.74)	0.68 (0.15–2.97)	5 (6.85)	0.65 (0.25–1.70)
No	475 (86.68)	21 (4.42)		19 (4.00)		48 (10.11)	
Intraoperative hemostasis use							
Yes	397 (72.45)	17 (4.28)	0.71 (0.31–1.62)	14 (3.53)	0.75 (0.30–1.90)	40 (10.08)	1.19 (0.62–2.29)
No	151 (27.55)	9 (5.96)		7 (4.64)		13 (8.61)	
Post-surgery hypertension							
Yes	113 (20.62)	7 (6.20)	1.45 (0.59–3.53)	4 (3.54)	0.90 (0.30–2.74)	20 (17.7)	2.62 (1.44–4.77)
No	435 (79.38)	19 (4.37)		17 (3.91)		33 (7.59)	

NA, not applicable; OR, odds ratio.

a One was a medial sural free flap, three were anteromedial thigh flaps, and one had missing data.

b Five patients received anterolateral thigh flaps. Three of them had tensor fascia lata flaps, and two had anteromedial thigh flaps. One patient received fibula and pectoralis myocutaneous flaps. Four patients used posteromedial thigh flaps combined with flaps from the pectoralis myocutaneous, posteromedial thigh, lower medial thigh, and a missing donor site.

^c^ It included superficial temporal veins mostly and some transverse cervical veins.

Thrombosis (OR 86.42, 95% CI 15.73–474.89), smoking (OR 49.44, 95% CI 1.28–>1000), PMT (OR 14.05, 95% CI 2.48–79.54), hematoma (OR 9.68, 95% CI 2.35–39.79), and younger age (OR 0.93, 95% CI 0.87–0.99) were associated with an increased risk of flap failure (Table 3).

**Table 3:** Multivariate logistic regression model for flap failure

	Failure (*n*=26)	
Variables	aOR (95% CI)	*P*
Thrombosis	86.42 (15.73–474.89)	<0.001
Hematoma	9.68 (2.35–39.79)	0.002
Age	0.93 (0.86–0.99)	0.029
BMI	1.03 (0.91–1.18)	0.634
Smoking	49.43 (1.28–>1000)	0.036
Hypertension	3.10 (0.95–10.13)	0.061
Free flap history		
Contralateral recurrence	1.51 (0.25–9.19)	0.655
Ipsilateral recurrence	1.20 (0.20–7.25)	0.839
Reconstruction time	2.21 (0.50–9.67)	0.293
Donor sites		
Fibula		
Forearm		
Tensor fascia lata		
Posteromedial thigh	14.05 (2.48–79.54)	0.003
Lower medial thigh		
Other	11.93 (0.51–278.07)	0.123
Double	1.30 (0.04–40.76)	0.882
Flap size	1.00 (1.00–1.01)	0.126
Recipient artery		
Facial artery	0.16 (0.01–4.55)	0.286
Carotid artery		
Superficial temporal artery	1.09 (0.22–5.31)	0.924
Other arteries	2.56 (0.30–22.15)	0.394
Vein number (Two)	0.48 (0.15–1.53)	0.216
External jugular vein	0.15 (0.02–1.19)	0.072
Superior thyroid vein	2.75 (0.42–18.03)	0.291

aOR, adjusted odds ratio.

Multiple reconstructions using a PMT flap (aOR 11.45, 95% CI 2.60–50.38, *P*<0.01) and the SThV as the recipient vessel (aOR 7.91, 95% CI 2.06–30.39, *P*<0.01) were independently associated with thrombosis (Table 4). Liver cirrhosis (aOR, 6.28; 95% CI 1.44–27.47, *P*=0.02), recurrent patients with an ipsilateral free flap reconstruction history (aOR, 0.14; 95% CI, 0.03–0.73; *P*=0.02), a fibula osteocutaneous flap (OR, 9.22; 95% CI 2.71–31.42, *P*<0.01), simultaneous double-flap reconstruction (aOR, 8.88; 95% CI, 1.80–43.81; *P*<0.01), the external jugular vein as the recipient vein (aOR, 0.22; 95% CI 0.08–0.61, *P*<0.01), and post-surgery hypertension (aOR, 2.77; 95% CI, 1.39–5.50, *P*<0.01) were independently associated with hematoma. Subgroup analysis revealed that postsurgical hypertension was associated with hematoma (*P*=0.028) in patients without hypertension (Table 5).

**Table 4:** Multivariate logistic regression models for thrombosis and hematoma

	Thrombosis (*n*=21)		Hematoma (*n*=53)	
Variables	aOR (95% CI)	*P*	aOR (95% CI)	*P*
Age	0.96 (0.91–1.01)	0.114		
Stage (late)	0.42 (0.14–1.28)	0.129		
BMI	1.10 (0.99–1.23)	0.066		
Pre-chemotherapy	0.24 (0.05–1.20)	0.083		
Alcohol			1.77 (0.87–3.61)	0.117
Diabetes			1.50 (0.75–3.01)	0.257
Severe CKD			1.23 (0.36–4.28)	0.741
Cirrhosis			6.28 (1.44–27.47)	0.015
Free flap history				
Contralateral recurrence			1.15 (0.38–3.46)	0.804
Ipsilateral recurrence			0.14 (0.03–0.73)	0.019
Reconstruction time	2.61 (0.77–8.88)	0.124		
Donor sites				
Fibula	1.92 (0.21–17.32)	0.561	9.22 (2.71–31.42)	<0.001
Forearm				
Tensor fascia lata	2.09 (0.21–20.94)	0.530	1.02 (0.19–5.56)	0.982
Posteromedial thigh	11.45 (2.60–50.38)	0.001	0.37 (0.04–3.31)	0.371
Lower medial thigh				
Other			1.88 (0.15–23.97)	0.626
Double	7.73 (0.52–114.80)	0.137	8.88 (1.80–43.81)	0.007
Flap size	1.01 (1.00–1.01)	0.067		
Recipient artery				
Facial artery			2.64 (0.88–7.95)	0.085
Carotid artery	3.30 (0.32–34.55)	0.319		
Superficial temporal artery	0.48 (0.08–2.94)	0.429	1.12 (0.37–3.44)	0.838
Other arteries	1.93 (0.42–8.83)	0.398	1.44 (0.39–5.36)	0.590
External jugular vein			0.22 (0.08–0.61)	0.004
Superior thyroid vein	7.91 (2.06–30.39)	0.003		
Post-surgery hypertension			2.77 (1.39–5.50)	0.004

aOR, adjusted odds ratio; CKD, chronic kidney disease.

**Table 5:** Hematoma according to post-surgery hypertension in hypertension subgroups

Subgroup		Hematoma				
Hypertension	With post-surgery hypertension	Post-surgery hypertension	No post-surgery hypertension	OR	aOR	*P* ^a^
Yes (*n*=216)	63 (29.16)	11/63 (17.46)	12/153 (7.84)	2.49 (1.03–5.98)	2.92 (0.94–9.05)	0.064
No (*n*=332)	50 (15.06)	9/50 (18.00)	21/282 (7.45)	2.73 (1.17–6.37)	3.42 (1.21–9.66)	0.021

aOR, adjusted odds ratio; OR, odds ratio.

^a^ Variables with *P*<0.2 in the univariate hematoma model were selected for adjustment, including alcohol consumption, diabetes, severe chronic kidney disease, cirrhosis, free flap history, donor sites, artery, external jugular vein, and post-surgery hypertension.

## Discussion

Thrombosis, smoking, posteromedial thigh flap usage, hematoma, and younger age were risk factors affecting flap survival in patients with HNC who underwent free flap surgery and received postoperative antithrombotic prophylaxis with PGE1, dextran-40, aspirin, and dipyridamole. The PMT flap and the SThV, which are used only after multiple reconstructions, are risk factors for thrombosis formation. Antithrombotic prophylaxis may need to be used with caution in patients with cirrhosis, fibula osteocutaneous flaps as donor sites, double-flap reconstruction, and postsurgical hypertension. This study collected detailed information from medical records to allow the simultaneous comparison of risk factors for flap failure, thrombosis, and hematoma.

Free tissue transfer has become the standard procedure for head and neck defect reconstruction after cancer ablation. The overall success rate of free flap reconstruction ranges from 90 to 98%^23–25^. In this study, the success rate was 95.3%, which is consistent with previous reports. The incidence rates of vascular thrombosis and hematoma in this study were 3.8% and 9.7%, respectively. All the patients in this cohort received antithrombotic prophylaxis. Compared with other studies in which antithrombotic prophylaxis therapies were not routinely administered, the thrombosis rates reported in our study were reduced to approximately half, but the hematoma rate was approximately doubled^26,27^. Notably, flap loss due to thrombosis was more than 4-fold greater than that attributable to hematoma. Although the effectiveness of prophylactic antithrombotic therapy in mitigating thrombosis remains a contentious issue in the field^28^, our data support that the use of antithrombotic prophylaxis agents could have a positive effect on flap survival.

Thrombosis is a major determinant of flap failure. Use of the PMT (or PAP) flap and the SThV as the recipient vessel were independent risk factors for thrombosis. In our group, the PMT flap was only used for recurrent cases when the ALT flap, the most commonly used flap, had been harvested from both thighs. Compared with other donor sites, the benefits of the PMT flap include the large amount of tissue available for flap reconstruction and a less visible scar location. However, the perforator pedicles of PMT flaps are relatively smaller in diameter and shorter in length than those of ALT flaps, and the limited number of healthy recipient vessels due to previous radiotherapy and multiple reconstructions for anastomosis might also explain the increased risk of thrombosis. Similarly, the use of SThV as a recipient vessel has been associated with thrombosis^29^. This association could be attributed to the smaller vascular caliber accompanying limited blood flow, which compromises flap circulation. SThV is usually of lower priority in recurrent cases, except for those in which regional vessels, cannot be found^30,31^. In our group, the SThV was chosen as the recipient vein because no other suitable larger vein was found in the recurrent neck region. Our observations highlight the importance of the selection of an alternative flap and recipient vein in the success of free flap reconstruction for recurrent patients.

Smoking has been suggested to result in flap failure by reducing tissue perfusion and delaying wound healing. Smoking can induce vasoconstriction, which decreases blood flow and impairs cell self-repair due to oxidative stress, lipid peroxidation, and inflammation^32,33^. In our study, smoking was identified as a risk factor for flap failure but not for thrombosis or hematoma. This observation suggests that the influence of smoking on cell repair dysfunction may be the major mechanism contributing to flap failure^32^. Consistent with the findings of a previous study, we found that smoking had a more significant effect on flap failure than did chronic hypertension^34^, which tended toward significance.

Younger age was associated with flap failure; however, the effect of age at failure was not high. This observation is consistent with those of previous studies^6,35,36^. Flap size has been associated with flap failure but was not in this study. Min *et al.* ^37^ reported that a large flap size could be associated with a higher flap failure rate due to inadequate flap perfusion. In our series, a larger flap harvested more than two perforators for enhancement. In accordance with the finding that two recipient veins tended to protect the flap from failure, our data support that recruiting more perforators in a larger flap to enhance circulation may improve flap survival.

The risk of hematoma as a complication can potentially be reduced by adopting antithrombotic regimens. Cirrhosis, ipsilateral recurrence, the use of a fibula osteocutaneous flap as a donor site, double-flap reconstruction, and postsurgical hypertension were identified as independent risk factors for hematoma. Cirrhosis impairs effective coagulation^38^. Patients with ipsilateral recurrence may have a lower risk of hematoma as neck dissection was previously performed. The fibula osteocutaneous flap is mostly used as a donor site in bone defect reconstruction. The fibular flap is known for its abundant blood supply. A longer duration of bone–involved reconstruction may increase the risk of hematoma and infection. The use of two donor sites, consisting of two flap reconstructions simultaneously, may also be associated with hematoma due to the complexity and increased duration of the surgery. Double flaps always require two separate recipient vessels, one for each flap, for anastomosis, which might have the potential to increase the flap failure rate. The EJV may be a good recipient choice. The EJV travels laterally to the sternocleidomastoid muscle, which provides a relatively stable foothold, and its subcutaneous location is also convenient for operation^39^. A larger EJV may increase venous outflow and contribute to less hematoma and failure^40^. In our study, the EJV tended to have a reduced risk of failure and hematoma. Additionally, hypertension after surgery was associated with hematoma formation. High blood pressure increases the risk of hematoma^41^. In this study, we found that post-surgery hypertension was more strongly associated with hematoma than chronic hypertension was. These findings indicate that blood pressure control is important for preventing hematoma. Given that post-surgery hypertension is often caused by pain, providing sufficient pain relief to decrease the risk of post-surgery hypertension may reduce the risk of hematoma. However, this hypothesis requires further validation.

This study has several limitations. First, this was a retrospective study; therefore, some data may be missing that may confound the results. Second, despite the large sample size in this study, the high success rate and salvage rate resulted in a small sample size for the outcome variables. Additionally, the ALT flap accounted for the vast majority of flaps (82.7%), and the superior thyroid artery accounted for 74.5% of all recipient arteries. The identified factors mainly apply to ALT flaps and less-represented radial forearm free flaps, which are also commonly used in some countries. Third, the study population was from a single medical center. Whether these results could be generalized to settings with different types of postsurgical care needs to be further validated.

## Conclusion

Antithrombotic prophylaxis has been found to be beneficial for patients given the impact of thrombosis on flap survival. The selection of donor flaps and recipient veins significantly affects the risk of thrombosis, hematoma, and flap failure. The findings of this study provide valuable insights into tailoring surgical planning and antithrombotic regimens to mitigate the risk of complications and flap failure in patients with head and neck cancer.

## Ethical approval

Institutional Review Board of Kaohsiung Medical University Hospital KMUHIRB-E(II)-20220204.

## Consent

Given the retrospective nature of the research, the IRB had granted waived inform consents for this study.

## Source of funding

The study was sponsored by Kaohsiung Medical University Research Foundation (KMU-M112026). The sponsor had no involvement in data collection, analysis and interpretation, writing of the manuscript, nor the decision to submit the manuscript for publication.

## Author contribution

P.H.H.: conceptualization, data curation, data analysis, writing original draft, editing drafts, and approval of the final article. H.Y.H.: conceptualization, data curation, editing drafts, and approval of the final article. W.Y.C.: editing drafts, and approval of the final article. Y.R.K. and Y.C.L.: conceptualization, resources, data interpretation, editing draft, approval of the final article.

## Conflicts of interest disclosure

The authors declare no conflicts of interest.

## Research registration unique identifying number (UIN)

ClinicalTrials.gov registry.

Unique ID/registration ID：NCT06371365.

Hyperlink to the registration: https://www.clinicaltrials.gov/study/NCT06371365?term=NCT06371365&rank=1.

## Guarantor

Yur-Ren Kuo and Ying-Chi Lin.

## Data availability statement

The datasets will be available upon reasonable request.

## Provenance and peer review

Not commissioned, externally peer-reviewed.
